# To chew or not to chew? Exploring the influence of scented chew toys on puppy chewing

**DOI:** 10.3389/fnbeh.2025.1602063

**Published:** 2025-06-20

**Authors:** Rituparna Sonowal, Nathaniel J. Hall, Anastasia C. Stellato

**Affiliations:** Department of Animal and Food Sciences, Texas Tech University, Lubbock, TX, United States

**Keywords:** chewing, mouthing, dogs, behavior, engagement, welfare

## Abstract

**Introduction:**

Chewing and mouthing are natural oral behaviors in dogs, particularly during puppyhood, yet owners report these as nuisance behaviors. The provision of appropriate enrichment items, such as scented chew toys, are often recommended to reduce these undesirable behaviors. Nevertheless, the influence of providing scented chew toys on chewing and mouthing behavior in puppies has not been investigated.

**Methods:**

We collected data on engagement levels in dogs (*N* = 29) with commercially available rubber chew toys during two 5-min sessions in an observation room, with each dog interacting with both toy types (non-scented, peanut butter-scented with squeaker) on separate days. Following the sessions, dogs were randomly assigned to be provided with either the non-scented (*N* = 15; control) and scented squeaker toy (*N* = 14) for 1 week in their household. Before and after the 1-week period, owners filled out an online questionnaire detailing how often their dog engaged in chewing and mouthing behaviors at home and they rated their agreement with various statements about their dog’s chewing behavior.

**Results:**

During observation sessions, puppies spent more time interacting with (*p* = 0.02) and sniffing (*p* < 0.0001) the peanut butter-scented squeaker toy in comparison to the non-scented toy. The frequency of owner’s prompting the dog to engage with the toy during the observation sessions was higher for the non-scented toy than the peanut butter-scented squeaker toy (*p* < 0.0001). Descriptive statistics reveal that owners in the non-scented group were more likely to agree that their dog’s mouthing or nipping was problematic in the second survey (61.5%, + 16%) compared to the initial survey (45.5%), while owners in the scented group were less likely to agree (41.6%, –28.4%) compared to the initial survey (70%). Owner reports suggest no changes in the frequency of dogs chewing on undesired items and mouthing or nipping on body parts during the 1-week period for either toy type.

**Discussion:**

The findings suggest that scent can enhance engagement with chew toys, and although chew toys did not influence owner perceptions, future research should evaluate the use of structured owner-implemented training strategies on mitigating unwanted chewing or mouthing behavior in dogs.

## 1 Introduction

Owned dogs exhibit behaviors (e.g., chewing, digging, chasing) that are intrinsically rewarding and highly motivating to perform, making these behaviors essential for their welfare (Mellor, 2016). Chewing is one of these highly motivated, natural behaviors that is linked to feeding, exploration of new environments and objects, jaw development, and learning (Seksel, 2008) and presents around 3 weeks of age during teething (Seksel, 2008). Chewing may also occur when adult dogs are experiencing negative emotional states, such as stress, boredom, frustration, high arousal, and anxiety (Arhant et al., 2021; Burn, 2017; De Assis et al., 2020; Lund and Jørgensen, 1999; Rooney et al., 2009). Without appropriate outlets for chewing, dogs may chew on inappropriate household items, which can pose a health risk if a dog ingests foreign objects (Melese, 1999) and be negatively perceived by the pet owner (Arhant et al., 2021). In addition to destructive chewing, puppies may also engage in mouthing or nipping behaviors that involve putting their mouth around a body part (Melese, 1999; Seksel, 2008), which can lead to human injury without appropriate intervention. These oral behaviors are often perceived as nuisance behaviors, and thus can impair the human-animal bond, lead to relinquishment or the use of aversive management techniques (Arhant et al., 2021). Thus, it is important to provide dogs with opportunities to chew and identify appropriate ways to mitigate unwanted chewing.

The recommended intervention to reduce puppy chewing includes removing the item or body part they are chewing or mouthing on and redirecting them to an appropriate item, which is commonly suggested to be a chew toy (Gazzano et al., 2008; Seksel, 2008). Recommended management/prevention strategies involve restricting access to household items that are either unsafe to chew or that owners do not want them to chew on (ASPCA, 2024; RSPCA, 2024). It is also recommended to provide various forms of enrichment both physical (e.g., exercise) and cognitive (e.g., puzzle feeders), and use positive reinforcement training to reward when dogs chew on appropriate items, like chew toys (ASPCA, 2024; RSPCA, 2024). Providing environmental enrichment through chew toys and safe, appropriate play with these items is recommended to reduce boredom and undesirable behaviors (e.g., destructive chewing and mouthing) and improve dog welfare (Burn, 2017; Melese, 1999; Wells D. L., 2004).

Chew toys have been shown to influence behavior in dogs, particularly in controlled environments like shelters and laboratories. In animal shelters, providing safe and durable chew toys or items has been considered an easy and effective enrichment strategy (The Association of Shelter Veterinarians, 2022), and guidelines for the care and management of laboratory dogs recommend providing enrichment items made of nylon and rawhide to support chewing behaviors (Prescott et al., 2004). Studies indicate that laboratory and shelter dogs showed increased interactions with chew toys (Nylabone, rawhide), noise-making toys (i.e., squeaky bone), and toys that are easy to chew (i.e., squeaky bone, plush teddy) compared to complex, indestructible toys (i.e., boomer ball) (DeLuca and Kranda, 1992; Pullen et al., 2010; Wells D., 2004).

Chew items have been suggested to be effective in reducing stress and anxious behavior when briefly left alone in a room in shelters (Flint et al., 2023), reducing plaque and tartar buildup (Arhant et al., 2021; Ketter et al., 2020; Logan, 2006) and reducing undesirable behaviors. Hubrecht (1993) found that when giving puppies chew toys, such as Nylabone and Gumabone, for 2 months, in a laboratory setting, puppies displayed fewer instances of inappropriate (destructive) chewing (Hubrecht, 1993). Notably, the puppies continued to engage with the toys over time without showing signs of habituation, indicating the lasting benefits of chew toy enrichment.

Enhancing engagement with chew toys is a key factor in ensuring that dogs interact meaningfully with enrichment items. The respondents of a survey-based study reported that chewing on items prevented boredom, had a calming effect on their dogs, served as an outlet for play behavior, and deterred their dog from chewing on household items (Arhant et al., 2021). They also reported that dogs preferred dried animal innards or meat and bone chew items over inedible or hard-chewing items (Arhant et al., 2021), likely due to their appealing scent (Murtagh et al., 2020) and association with food (Butler and du Toit, 2002; Forsyth et al., 2014). The strong olfactory sense of dogs encourages exploration, and scented toys can stimulate both engagement and environmental exploration (Amaya et al., 2020; Boonhoh et al., 2024; Kokocińska-Kusiak et al., 2021; Murtagh et al., 2020).

Given the potential of food-scented enrichment toys to influence behavior and improve welfare, our study aims examine how scented chew toys may impact the behavior of pet dogs, particularly puppies. The current study therefore assessed the level of engagement between scented squeaker chew toys and non-scented chew toys in young puppies, and whether scented chew toys can decrease the chewing and mouthing behavior of young puppies compared to non-scented chew toys.

## 2 Materials and methods

The study protocol was reviewed and approved by the Institutional Animal Care and Use Committee (AUP#2023-1439) and the Institutional Review Board (IRB#2024-113) at Texas Tech University.

A total of 29 dogs were recruited online using social media, where dog owners were given details on participation. To be eligible to participate, the dog owners needed to be 18 years of age or older, residing within Lubbock County and their dogs had to be between 3 and 10 months of age, healthy, and received their first round of vaccinations.

During recruitment, an online survey was provided via Qualtrics^®^ to dog owners to collect dog demographic information (i.e., age, sex, breed, health status) and information on their dog’s chewing behavior. Owners were asked to report on the prevalence of their dog causing damage to off-limit household items via chewing (No, Yes—minor damage, Yes—major damage). Off-limit items were defined as items that they would not want to their dog to chew on. Also, the frequency of their dog chewing on off-limit items, toys, and mouthing on body parts were determined using a Likert scale [Never, Rarely (once total), Occasionally (2–3 times), Regularly (once/day), Frequently (2–3×/day), Very Frequently (> 3×/day)]. Owners were also asked to report their level of agreement (on a scale from Strongly Agree to Strongly Disagree) with the following statements: Chewing toys help minimize any unwanted chewing on off-limit items, My dog readily chews on the toys I provide them, and My dogs chewing/nipping/mouthing behavior is a problem.

### 2.1 Assessing the level of engagement between scented squeaker and non-scented chew toys

All testing took place in an observation room (19.6 ft by 15.8 ft) over two non-consecutive days, with an average of 3 days between visits. To assess the level of engagement with different rubber chew toys, two commercially available products were selected for comparison: a non-scented toy (Extreme Goodie Bone, rubber, Kong^®^) and a scented toy with a squeaker (Teething bone, peanut butter-scented, rubber, Playology^®^). The squeaker in the scented chew was located in the center of the toy and both toys were made of a durable rubber material. The non-scented toy served as the control and the scented squeaker toy served as the treatment. To assess toy preferences, each dog participated in two separate play sessions, each held on a different day. In the first session, the dog was randomly assigned to one of the two toy types by using a random number generator (random.org), such that an odd number was assigned to the non-scented toy an even number was assigned to the scented toy, and the dogs were allowed to engage freely with the toy. In the second session, the dog received the other toy type that was not provided during the first session. This setup allowed for an individual assessment of each dog’s interaction with each toy.

**FIGURE 1:**
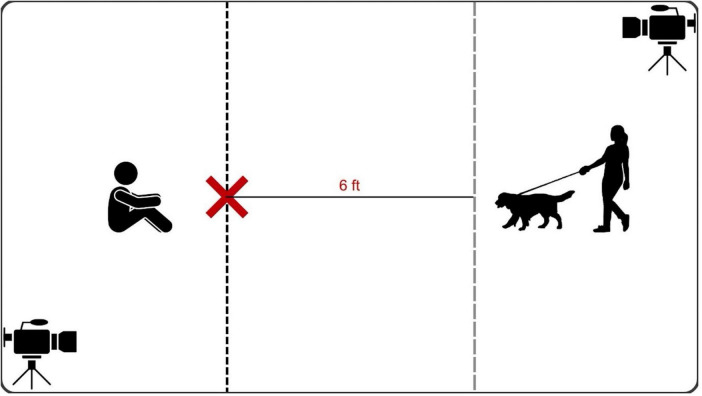
A schematic diagram of the layout of the testing session with the scented and non-scented toy available on the x-marked area for the dog to freely engage with.

Upon the participant’s arrival at the facility, informed consent was obtained before guiding them to the observation room. Dogs were given approximately 3–10 min to acclimatize to the room, with the length of the period determined based on their behavior. In one case where the dog showed signs of fear toward the handler, the acclimation period lasted the full 10 min, concluding once the dogs appeared comfortable (e.g., no longer exploring the environment and/or reduced general movement), with their owner and handler present. After acclimation, the researcher briefly entered the room to provide the assigned toy to the handler and turn on the cameras before exiting. The handler provided the toy to the owners, and then held the dog’s leash, which was provided by the owner. The owners were instructed to engage their dogs with the toy by calling, showing, or squeaking the toy and allowing the dog to smell the toy before proceeding to place it down in the marked area (Figure 1). Once the handler dropped the leash, the 5-min timer was started to initiate the observation period. During the 5 min, the dog was given the opportunity to freely explore and interact with the toy. If the dog did not interact with the toy within 1 min, the handler prompted the owner to re-engage the dog with the toy. The session was concluded at the completion of the 5-min period. Owners were instructed to schedule the next session within 1 week. The same procedure was repeated during the second play session with the other assigned toy. Owners and handlers were blinded to toy types and the study hypothesis. All testing was video recorded using two video cameras (Sony HDR-CX405 HD Handycam), one positioned near the handler and the dog, and the other positioned behind the owner. Each session took approximately 15 min.

### 2.2 Assessing the influence of chew toys on owner-reports of puppy chewing behavior

After completion of the second play session, the dogs were randomly allocated to two treatment groups, scented (*n* = 15) and non-scented (*n* = 14), in a randomized block design, with dog age (3–7 months, 8–10 months) balanced between groups. Depending on their assigned group, dogs were sent home with their assigned toy for 1 week (7 days). All dogs had existing chew toys in their home and dog owners were instructed to not remove the toys that were already present in their homes and multi-house dog owners were specifically instructed to ensure toy access to the participating dog. Owners were advised to separate the testing toy from the other household dogs and those owners who housed their puppies in a crate were also instructed to place the toy inside their crate when not present at home. After completion of this phase, owners were given a secondary survey to complete after the 1-week period (on the 8th day). The survey included identical questions from the initial recruitment survey regarding the prevalence and frequency of their dog chewing off-limit items and owner perceptions of their dog’s chewing behavior.

### 2.3 Statistical analysis

During observation sessions, continuous sampling was used to record the frequency, and duration of toy-directed behaviors displayed by participating dogs and the number of prompts given by the owner during each 5-min play session. Behaviors assessed were chewing, sniffing, licking, and pawing. Also, the duration of each of these behaviors were summed to identify the total interaction time with each toy type. Behavioral scoring was completed by one observer using Observer XT 16 (Noldus Information Technology Inc., Wageningen, Netherlands). The intra-rater reliability for the behavioral assessment was assessed and Cohen’s kappa of 0.89 indicated high agreement.

All statistical tests were conducted in R Studio (R Core Team, 2013). Bar plots were created using the ggplot2 package in R (Wickham, 2011) and the R ColorBrewer package was utilized to color code certain graphs (Neuwirth and Neuwirth, 2014). Mixed regression models were created to evaluate the association between toy type (scented squeaker and non-scented) and dog’s age with the following outcome variables: 1) total interaction time (in seconds) 2) chewing (in seconds) 3) sniffing (in seconds), 4) pawing (categorized as either yes or no), and 5) frequency of owner’s prompting their dog to engage with the toy. Linear regression models were used to interpret total interaction time, chewing, and sniffing, a logistic regression model was developed for pawing behavior, and a Poisson regression model was used to assess the number of owner prompts. For all models, the dog was included as a random effect. The models were fitted using the *lme4* package (Bates et al., 2015). The *lmerTest* package was used to calculate the respective *p*-values for each model (Kuznetsova et al., 2017).

To evaluate whether there were any differences in the owner perceptions of the frequency of their dog’s chewing behavior from the end of the 1-week period (second survey) compared to baseline (initial survey), Wilcoxon sign rank paired tests were used. Descriptive data was used to report owner’s level of agreement on statements regarding their dog’s chewing. For ease of interpretation, agreement with the provided statements was categorized as “Agree” (including Strongly Agree and Agree) and “Disagree” (including Strongly Disagree and Disagree), and Neither Agree/Disagree was excluded due to few responses and its limited interpreted value. For ease of interpretation regarding the frequency of chewing on toys provided from the study, the Likert scale response (Never, Rarely, Occasionally, Regularly, Frequently, and Very Frequently) were categorized into two groups, “More Frequent” (including Regularly, Frequently, and Very Frequently), and ‘Less Frequent’ (including Never, Rarely, and Occasionally). Additionally, in the second survey owners were asked to recall if they noticed any changes (either no change, more chewing, less chewing) in their dog’s chewing behavior over the 1-week period. Owners who reported their puppy does not chew on off-limit items (*n* = 2) or mouth/nip on body parts (*n* = 4) in both surveys were excluded from the Wilcoxon analysis. Thus, a total of 27 dogs were included for assessing changes in chewing off-limit items and 25 dogs were included for assessing changes in mouthing or nipping on body parts.

## 3 Results

### 3.1 Descriptives

A total of 29 dogs (13 males, and 16 females) were recruited and included in the analysis. Ages ranged from 4 to 10 months, with a mean age of 6.3 months. A total of 14 breeds were represented across 29 dogs. Of these, the scented squeaker toy group included: American Bully (1), Golden Doodle (1), Golden Retriever (3), Labrador Retriever (1), Mixed (7), and Poodle (1). The non-scented toy group included: Australian Shepherd (3), Basenji (2), Bolognese (1), Boston Terrier (1), Cockapoo (1), Doodle (1), Golden Doodle (1), Great Pyrenees (2), Mixed (2), and Schnauzer (1).

**FIGURE 2:**
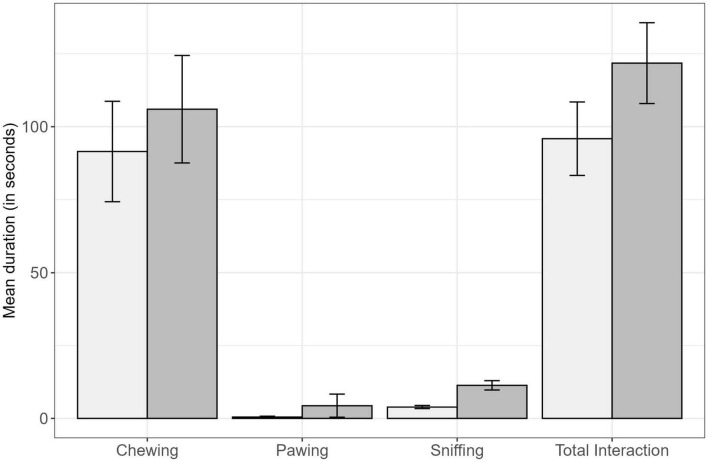
Displaying the duration (95% CI) of behavior and total interaction performed by dogs (*N* = 29) when engaging with the non-scented toy (light gray) and scented squeaker toy (dark gray).

The dogs spent more time (average time in seconds ± SE) interacting with the scented squeaker toy (122.2 ± 12.42) compared to the non-scented toy (95.8 ± 11.18; Figure 2). Chewing was also the most common toy-directed behavior displayed by all dogs compared to sniffing and pawing during the observation sessions (Figure 2). Only one dog was observed to squeak the scented squeaker toy during the observation sessions. During the 1-week period, 7 owners reported hearing their dog squeak the toy occasionally, 5 owners reported not hearing any squeaking, and 2 owners did not respond to the follow-up question.

#### 3.1.1 Owner perceptions of their dog chewing behavior at home

**FIGURE 3:**
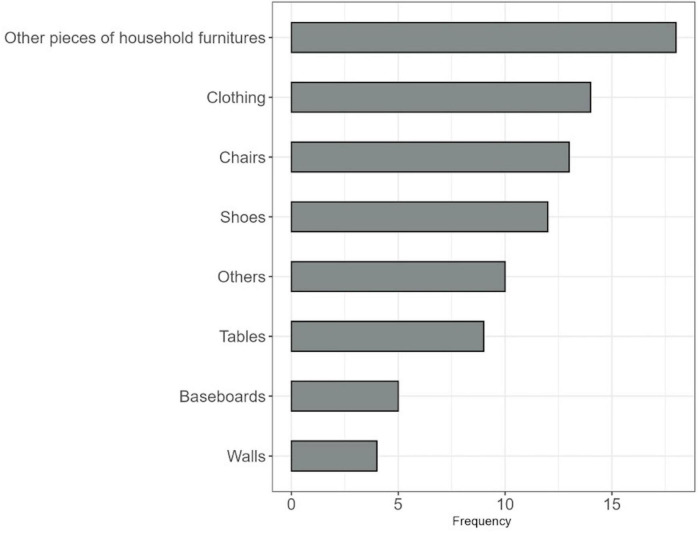
Displaying the number of participants who reported the number of household items that their dog chews on, as reported from the initial survey, with other pieces of household furniture including clothing, chairs, shoes, tables, baseboards, and walls.

In the initial survey, the majority of dog owners reported that their puppy causes minor damage to off-limit household items (65.51%, 19/29), while 20.68% (6/29) reported no damage and only 13.79% (4/29) reported major damage. The most frequently reported items to be chewed on were other pieces of household furniture (e.g., couch legs, pillows, carpet) and clothing (Figure 3).

**FIGURE 4:**
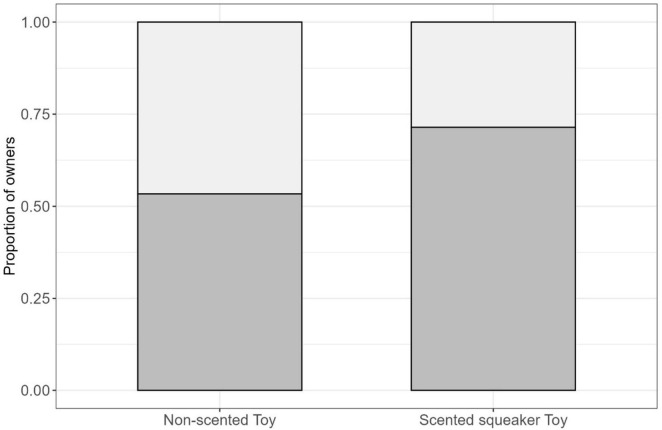
Displaying the proportion of participants in the non-scented group (*n* = 15); and scented group (*n* = 14) that reported the frequency (light gray = less frequent; dark gray = more frequent) of their dog chewing on the provided toys during the 1-week observation period.

When owners were asked if they noticed any changes in their dog’s chewing behavior on off-limit items after the toys were assigned, less chewing on off-limit items was relatively evenly reported by owners in the scented group (50%, 7/14) and in the non-scented group (46.7%, 7/15), with one owner in the non-scented group reported more chewing. Over a 1-week period, owners reported how frequently [Less frequent (Never, Rarely, and Occasionally), More frequent (Regularly, Frequently, Very Frequently)] their dogs chewed on the toy provided in the study. Among those who received the scented squeaker toy, 71.4% (10/14) of owners reported more frequent chewing, while 28.6% (4/14) reported less frequent chewing. In contrast, among those who received the non-scented toy, 53.3% (8/15) of owners reported more frequent chewing, while 46.7% (7/15) reported less frequent chewing (Figure 4).

#### 3.1.2 Owner agreement with statements regarding their dog’s chewing behavior

Owners were asked to report their level of agreement to statements related to their dog’s chewing behavior during the initial and second surveys. Regarding whether their dog readily chews on toys the owners regularly provide them, agreement increased in both groups from the initial to the second survey (Table 1). Regarding whether chew toys can help minimize chewing on off-limit items, all owners in the scented group remained in agreement, but agreement slightly declined in the non-scented group. Regarding whether their dog’s mouthing/nipping is a problem, owners in the scented group reported decreased agreement, and owners in the non-scented group reported an increase in agreement from the initial to the second survey (Table 1).

**TABLE 1:** Displaying changes in participating dog owner agreement/disagreement with statements regarding their dog behavior from the initial survey to the second survey after the completion of the 1-week observation period.

		Scented			Non-scented		
		Initial survey	Final survey	Change	Initial survey	Final survey	Change
Mydog readily chews on toys that I provide them	Agreement	13/14 (92.86%)	13/13 (100%)	+7.1%	10/12 (83.3%)	11/12 (91.6%)	+8.3%
	Disagreement	1/14 (7.14%)	0/13 (0%)	–7.14%	2/12 (16.7%)	1/12 (8.3%)	–8.4%
Chew toys help minimize chewing off-limit items.	Agreement	14/14 (100%)	13/13 (100%)	0%	12/13 (92.3%)	12/14 (85.7%)	–6.6%
	Disagreement	0/14 (0%)	0/13 (0%)	0%	1/13 (7.7%)	2/14 (14.3%)	+ 6.6%
Mydog’s mouthing/nipping is a problem	Agreement	7/10 (70%)	5/12 (41.6%)	–28.4%	5/11 (45.5%)	8/13 (61.5%)	+16%
	Disagreement	3/10 (30%)	7/12 (58.3%)	+28.3%	6/11 (54.5)	5/13 (38.5%)	–16%

### 3.2 The difference between toy-directed behavior between each toy type

The regression results indicate that the total interaction time (in seconds) was influenced by toy type, with dogs interacting more with the scented squeaker toy than with the non-scented toy (*p* = 0.02; Table 2). There were no detected differences in time spent chewing between the toy types (*p* = 0.18; Table 2), and dog age was not associated with the total interaction time (*p* = 0.28) or chewing behavior (*p* = 0.20; Table 2). Dogs spent more time (in seconds) sniffing the scented squeaker toy compared to the non-scented toy (*p* < 0.0001; Table 2).

**TABLE 2:** Mixed linear regression models assessing the influence of toy type (scented toy and non-scented toy) and dog age on total interaction time (in seconds), sniffing (in seconds) and chewing (in seconds) with dog (*N* = 29) included as a random effect.

Variables		Estimates^a^	CI (95%)^b^	*P*-value
**Total interaction time (Model 1)**				
Toy type	Scented vs. non-scented	26.35	5.26, 47.44	**0.02**
Age		8.40	-6.38, 23.18	0.28
**Chewing interaction time (Model 2)**				
Toy type	Scented vs. non-scented	14.95	–6.79, 36.68	0.18
Age		10.05	–4.81, 24.91	0.20
**Sniffing interaction time (Model 3)**				
Toy type	Scented vs. non-scented	7.43	4.72, 10.15	**<0.0001**
Age		-1.06	–1.86, –0.26	**0.01**

^a^Coefficient estimates based on the output of regression models. ^b^95% confidence intervals. Bolded values indicate significance at *p* < 0.05.

The frequency of owner prompts was higher for the non-scented toy than for the scented squeaker toy (*p* = < 0.0001; Table 3). Dog age was negatively associated with sniffing, where the duration of sniffing reduced as the dog’s age increased (*p* = 0.01; Table 3). Also, the odds of pawing behavior was 39.1 times higher with the scented squeaker toy compared to non-scented; however, the effect was not significant (*p* = 0.18), and age did not influence pawing behavior (*p* = 0.82; Table 3).

**TABLE 3:** Mixed regression models assessing the influence of toy type (scented toy and non-scented toy) and dog age on pawing and frequency of owner prompts with dog (*N* = 29) included as a random effect.

Variables		OR^a^	CI (95%)^b^	*p*-value
**Pawing (model 4)**				
Toy type	Scented vs. non-scented	39.1	0.18, 8690.62	0.18
Age		0.87	0.28, 2.73	0.82
		**IRR^c^**	**CI (95%)**	***p*-value**
**Frequency of owner prompts (model 5)**				
Toy type	Scented vs. non-scented	0.68	0.56, 0.82	**<0.0001**

^a^Odds ratio based on the output of the logistic regression model. ^b^95% confidence intervals. ^c^Incidence rate ratio based on the output of the Poisson regression model. Bolded values indicate significance at *p* < 0.05.

### 3.3 Changes in the frequency of dog chewing behavior from initial to final survey based on owner reports

Owners reported no observed changes in their dogs’ chewing on off-limit items from the initial to the second survey in the non-scented group (Wilcoxon sign rank test; V = 28.5, *p* = 0.50) and scented group (Wilcoxon sign rank test; V = 10, *p* = 0.53). Owners reported no observed change in chewing on toys that the owner generally provides (outside of the experimental toys provided), from the initial to final survey in the non-scented group (Wilcoxon sign rank test; V = 25, *p* = 0.83) and in the scented group (Wilcoxon sign rank test; V = 10, *p* = 0.08). Also, owners reported no observed changes for mouthing or nipping on body parts (e.g., hands, arms) from initial to final survey in the non-scented group (Wilcoxon sign rank test, V = 10.5, *p* = 0.30) and also in the scented group (Wilcoxon sign rank test; V = 3.5, *p* = 0.16).

## 4 Discussion

This study aimed to investigate the influence of scented chew toys on chewing behavior performed by puppies in household settings. Based on the time spent interacting, puppies engaged more with scented squeaker chew toys and needed to be prompted more frequently by their owner to engage with the non-scented, non-squeaking toy, suggesting that dogs might be less interested in interacting with the non-scented toy. Also, despite chewing being a frequently displayed behavior when engaging with both chew toys, dogs engaged in more sniffing behaviors with the scented squeaker toy compared to the non-scented toy, with older dogs sniffing less. As exploratory behaviors, like sniffing, toward novel objects have been reported to decrease with age (Rosado et al., 2012), this might explain why sniffing behavior was negatively associated with dog age in our study.

Although there were no significant differences in the frequency of the dog’s mouthing and chewing behaviors across the 1-week period based on owner reports, a trend emerged from owner perceptions. Regarding the perception of chewing on off-limit items, owners reported that their dog displayed less chewing on off-limit items after assigning the toys, irrespective of the toy type. This suggests that chew toys, regardless of scent, can be effective at improving owner perceptions of their dog’s chewing behavior. Also, more owners in the scented group reported that their dogs chewed on the toy provided and they perceived their dog’s mouthing or nipping as less problematic compared to owners in the non-scented group.

Previous research showed that dogs can greatly benefit from scented enrichment due to their strong sense of smell (Boonhoh et al., 2024; Murtagh et al., 2020) and are more likely to chew on toys that make noise (DeLuca and Kranda, 1992; Hubrecht, 1993). Also, it has been previously discovered that out of all chewing materials, dogs show a clear preference for edible materials and this was suggested to be elicited from the odor associated with these materials (Arhant et al., 2021). Thus, it is possible that the peanut butter scent served to enhance the dog’s interaction with the scented squeaker toy, as it may have made it more engaging and enriching for them. The scented squeaker toy not only had a strong peanut butter scent, but it also contained a squeaker, which the non-scented toy lacked. This additional feature could have made the toy more engaging and possibly influenced the dogs’ behavior, regardless of the scent, thereby introducing bias. The squeaker was only activated by one dog during our observation sessions, and a few dog owners in the scented group reported that their dog managed to squeak the toy during the 1-week period. This is likely due to the central placement of the squeaker and the tough exterior, and owner reports and direct observations indicated that most dogs primarily chewed on the toy’s edges. Despite this, it is possible that the presence of the squeaker influenced findings and we cannot definitively conclude that the observed differences were due solely to scent. Future studies should aim to isolate these variables by using toys that are identical in all features except for scent.

The lack of observed changes in the frequency of the dogs’ chewing and mouthing behaviors, could be a result from the small sample size. The current data contrasts with a previous study by Hubrecht (1993), which investigated puppies housed in laboratory conditions and found that Nylabone chew toys were effective in reducing furniture destruction. While this previous study used controlled laboratory data, and the current study relied on owner reports, one possible explanation for the lack of observed changes in chewing behavior in the current study could be the absence of specific inclusion criteria regarding chewing or mouthing behavior during the recruitment process for the puppies. The only specific criterion was the age range of 3–10 months. Due to the lack of inclusion criteria during the recruitment process for causing damage to off-limit items, our sample included a majority of puppies that caused minor or no damage to off-limit items and only a small proportion that caused major damage. Although we excluded owners who reported no inappropriate chewing, those who reported only minor damage to off-limit items may not have exhibited changes because their dog did not have significant chewing issues. This limited variation in chewing behavior likely led to floor effects, making it difficult to detect observable changes. Within this pool of participants, where the majority reported no to minor damage to off-limit items, the effect size for the frequency of chewing on off-limit items in the scented group was low (0.26), and power analysis indicates a sample of 124 puppies would have been needed to detect this difference. Thus, attaining a larger sample of dogs with more pronounced chewing issues might support the ability to detect observable changes in owner perceptions of their dog’s chewing behavior at home. Additionally, there was no ‘true control’ group which received no intervention; thus, it remains unclear whether the observed changes were due to the toys themselves or the study process itself, including the use of questionnaires and the sessions with owners, influencing owner interactions with their dogs and their perceptions of the dogs’ chewing behavior. Future research should also explore additional factors, such as owner management (e.g., type of enrichment provided, response to chewing behaviors), household factors (e.g., access to off-limit items), and presence of the same type of chew toy with and without scents to further explain owner perceptions and puppy chewing behaviors. As this area of research is relatively underexplored, future studies are needed to understand the effectiveness of the long-term provision of scented chew toys as an intervention strategy where the issue is more prevalent and its effects on overall dog behavior and welfare.

## Data Availability

The data publicly available at: https://github.com/ritzp30-dot/puppy_study.git.
